# Errors in Domestic Medicine

**Published:** 1884-06

**Authors:** 


### ERRORS IN DOMESTIC MEDICINE.

BY AN OLD PRACTITIONER.

EVERY woman is a physician at heart, and nothing is more refreshing than to sit and listen to two ladies in confidential medical conversation respecting the merits of their favorite nostrums. It is to them that homeopathy especially appeals. What more delightful spectacle can be found than that of a fair amateur

'doctress' with her book, her case of phials and little gold spoon, dispensing globules to her family, to her servants, to her neighbors, to any one and every one; and to enjoy at the same time the sweet reflection that she is not doing a particle of harm! Nevertheless, there are some not unfrequent mistakes in the application of so-called household remedies, excellent in themselves; and to call attention to these, and to a few popular fallacies on the subject of health and disease, is the object of the present paper.

Let us commence with that finest of domestic institutions, the poultice—bread, linseed, or mustard—soothing, fomenting, or stimulating, according to circumstances. There are few remedies in the pharmacopœia, of wider beneficial application in surgery and medicine than this; yet terrible mischief often follows its injudicious use. A man has a cough, or his child wheezes with a 'tightness on the chest,' and on goes a poultice straightway. So far, so good; in all probability they wake up next morning greatly relieved. But the father is off to his daily business, and the child runs about and plays as usual, while—since they feel so much better—neither takes any precaution, by extra clothing or otherwise, to guard against the consequences of the poultice itself. The skin and subjacent tissues have been rendered lax by the heat and moisture, the blood-vessels are dilated, and the circulation of the part increased; to use a common expression, the 'pores' are open, and there is thus a tenfold liability to catch cold, especially in winter-time, when these things most frequently happen. Ordinary colds which are said to have 'run' into congestion of the lungs, bronchitis, or pneumonia, may often be traced to their serious or fatal termination through the *undefended* use of a poultice.

It should be borne in mind that a common poultice—such as is made of linseed meal or bread—is merely a vehicle for the application of damp heat—a continuous fomentation, in fact—and has no specific curative action. A muslin bag filled with bran, or flannels dipped in hot water, have precisely the same effect, but are not so conveniently employed, as they have to be more frequently renewed. A poultice is homogeneous in consistence throughout; just so wet as to permit of its retaining the mould of the cup when turned out, but not wet enough to exude water by its own weight when lightly applied. A *hot* poultice should never be allowed to remain on after its outer part is less than the temperature of the blood, nor must it get dry and caked. As a general rule it may be said that bread makes a better cataplasm than linseed meal, but requires to be changed oftener. There are, of course, special medical reasons in occasional cases for the preference of the one or the other, but such instances rarely come within the scope of this article. Well-mashed carrots make a capital soothing application, and a poultice composed of tea-leaves is, owing to its slight astringent action, generally suitable when one is required about the region of the eye. An abominable mixture of soap and sugar is very popular as a local remedy in some parts of England, and is credited with 'drawing' properties. On the other hand, it is good to know that the old-fashioned liniment of hartshorn and oil is one of the best embrocations ever invented under ordinary circumstances, and that therapeutical research amongst all the drugs that the vegetable and mineral kingdoms afford, has never discovered an improvement on salt and water as a gargle for simple sore throat.

What British home would *be* a home without its little roll of sticking or court plaster? How often is it that little tearful eyes look mistily down on a poor scratched finger, held carefully out in the other hand, as if there was some danger of its coming off, while mamma cuts a thin yellow strip and wraps around the injured member with comforting words, all lamentation being temporarily reduced to an occasional sob in the interest of the operation. That the sticking-plaster exercises a fine moral effect in such a case, there can be no doubt; but I fear there is as little doubt that it often does more harm than good from a physical point of view, and this arises from a fallacious belief in it as a healing agent. The only real service that sticking-plaster does is to hold two cut surfaces together while Nature's process necessary for their union is being completed, acting for a slight wound as stitches do in a deep one. But to cover abrasion or raw surface with it is worse than useless, as it only irritates it. The plea is often advanced that it serves to keep the dust and dirt off. A bit of wet linen rag, however, would be far better for that purpose.

Most of the ordinary household cures for chilblains are well enough in their way, but an unfortunate mistake is often committed in applying certain of them, which are fit only for the chilblains in their early stage, to broken ones, setting up thereby great inflammation and producing painful sores, A broken chilblain is a little ulcer, and must be treated as such. As for the thousand-and-one remedies in vogue fcr corns, it is wonderful that they should exist at all, since nine people out of ten could cure their own without any application whatever, by wearing properly fitting boots and shoes. It is irregularity of pressure which creates corns; boots which are too big being as productive of the tiny torments as tight ones. A wet rag covered with oiled silk—to retain the moisture—and bound around the corn, is one of the best cures.

A very common but reprehensible practice is that of holding a burn as close to the grate as possible, to 'draw the fire out'—not out of the fireplace—but from the injured part. It is quite feasible to conceive that such a proceeding may give ease by deadening sensation in some instances; but it by no means follows that it does good or expedites recovery—indeed, we shall see that in such a case the loss of sensation really proves further damage to the tissues. Burns have been divided by surgeons into six classes: (1) Simple scorching, sufficient only to redden the surface. (2) Blistering; the cuticle raised and forming little bladders of water. (3) The skin denuded of its cuticle. This is the most painful stage of all, as it leaves the nerve-ends exposed. (4) Destruction of the entire thickness of the skin; painless or nearly so, because the sensitive nerve-bulbs are destroyed. (5) Destruction of all the soft parts; and (6) charring of the bone—two conditions very difficult to imagine as co-existent with any remnant of life. It can thus be readily understood how a burn of the third order of magnitude converted by additional heat into the fourth, and temporary relief from pain purchased by transforming a trifling injury into a serious one, liable to be followed by severe illness and permanent deformity. A most mysterious cause of death after burns is the ulceration and bursting of a certain blood-vessel in the stomach. The connection between the two has never been discovered. People talk about this or that being good for a burn, but not for a scald, or *vice versa;* but practically no distinction is to be drawn between the two, further than that, as we know the highest temperature of water, we know the utmost limit of injury in a scald, whereas there is no limit to the possibilities a burn. To keep the air from both is the main object in treatment. Cook, who generally appears on the scene of the disaster with her flourdredge, is a very efficient surgeon for burns and scalds of the first degree—this little scientific technicality will comfort the sufferer marvellously; but where the skin is raised or broken, something of an oily nature—Carron oil, for instance—should be substituted. Cover it up with lots of cotton-wool, as though you wish to keep it as warm as possible; and, mind, no soap and sugar on any account!

What is the origin of the popular idea that the finger-nails are poisonous to a wound? It does not do a wound much good to scratch it, or indeed touch it, but that is no reason why those useful little shields of our finger ends should be so libelled. Whence comes the notion that to pierce a girl's ears and compel her to wear earrings improves her eyesight? Possibly this may have arisen from the fact that medical men sometimes put blisters behind the ears as counter-irritants, to relieve some chronic ophtalmic disorders. Why is a glass of hot rum-and-water with a lump of butter in it not only familiarly prescribed for but familiarly swallowed by catarrh-afflcted mankind? Speaking of colds generally, we may remark in passing that treacle posset, hot gruel, putting the feet in mustard-and-water, &c., are all capital things, but that they effect only the one object of inducing perspiration. There is nothing specifically curative about any of them. It is a mistake, however, to give spirits, negus, or any alchoholic fluids in influenza colds where there is much congestion of the mucous membranes, as it increases the incidental headache.

'Is the bone broken, or only fractured, doctor?' is an anxious question often asked apropos of an injured limb. Broken and fractured are synonymous terms in surgery, my dear madame—it is always a lady who asks this—but I think I know what you mean. A fully developed bone is rarely cracked—nearly always it snaps in two pieces—but the soft cartilaginous bones of children sometimes sustain what is called a 'green-stick fracture,' a name which almost explains itself, meaning that the bone is broken through part of its thickness, but not separated, as happens with the green bough of a tree. Many people have a totally erroneous idea, when an arm or leg is badly bruised only, that it would be better if it were broken. Right across the muscle, too!' implies, that an injury has been received across the the upper arm in the region of the biceps, that being the only 'muscle' which is honored by general public recognition. How many people know that what they call their flesh, and the lean part of meat, is nothing but muscles, the pulleys by which every action of the body is performed? Common mistakes lie in trying to 'walk off' rheumatism, sprains, and other things which should be kept entirely at rest; and in squeezing collections which have burst or been lanced, with a view to hasten their healing by the more speedy emptying of their contents.—*Chambers's Journal.*

MENTHOL PENCILS.—We wish to call attention to the advertisement of Parke, Davis & Co., on the fourth page of the cover of this number of the JOURNAL. The use of the pencils is there fully explained.

*Extract from the London Medical Press and Circular of Jan. 30th,* 1884.—The difficulty of so disguising the taste of cod-liver oil as to make it palatable to invalids and children, has long stimulated the ingenuity of pharmacists to devise a mode of preparing it so that the distinctive taste should be lost. Some of these are more successful than others, but the preparation before us, which we have tested in actual practice, possesses all the advantages claimed for it, and we are glad to note that it is gaining ground in this country as a safe and efficient remedy. This Scott's Emulsion is of American origin, being made by Messrs. Scott & Bowne of New York. Pure cod-liver oil exists in it to the amount of 50 per cent., while each ounce of the emulsion contains six grains of hypophosphate of lime and three grains of the same salt of soda, the tissue-stimulating properties of the mixture being thus incalculably increased. Moreover, it is almost pleasant to take, and possesses none of the objectionably qualities of the oil itself.

Sample of the above preparation will be sent to any reader of this journal who will send their post-office and express addresses to SCOTT & BOWNE, 103 Wooster St., New York.

